# Endometriosis in the rectum accompanied by hemorrhoids leading to diagnostic pitfalls: a rare case report

**DOI:** 10.1186/s12905-018-0615-z

**Published:** 2018-07-04

**Authors:** Xiuying Shi, Chuifeng Fan

**Affiliations:** Department of Pathology, First Affiliated Hospital and College of Basic Medical Sciences, China Medical University, Shenyang, 110001 China

**Keywords:** Endometriosis, Rectum, Hemorrhoids

## Abstract

**Background:**

Hemorrhoid is a common anorectal disease. Hemorrhoids accompanied by endometriosis are unusual. As endometriosis in the rectum may mimic many other diseases, including cancer and inflammation, its diagnosis may be difficult, especially when it is combined with other diseases.

**Case presentation:**

Here, we present a rare case of a patient with hemorrhoids accompanied by endometriosis in the rectum. The endometriosis mass was detected by digital rectal examination and CT scan and confirmed by pathological examination. The mass was approximately 0.8 cm × 0.6 cm and located in the muscularis and submucosa of the rectum 8 cm from the anus.

**Conclusions:**

In this case, hemorrhoid is a common disease of rectum and anal canal. However, when it is complicated by another rare disease, the rare one can be easily neglected because of the existence of the common one, especially when the two diseases have similar lesions or symptoms. We suggest that strict physical examination, such as the digital rectal examination in the current case, is critical for correct disease diagnosis.

## Background

Endometriosis mainly affects females of child-bearing age [1]. Endometriosis in the intestine mostly involves the rectum and sigmoid colon [2]. Although it is not a very rare disease, its diagnosis may be very difficult because its presentation may mimic that of many other diseases, such as cancer and inflammation [2, 3]. That is, its symptoms and imaging findings are not specific and are similar to those of these diseases. In the absence of experience, the lesion may even be misdiagnosed as adenocarcinoma under the microscope, especially on fast frozen section examination. In contrast, in some rare conditions, other lesions could be mistaken as endometriosis. Weng reported an unusual case of rectum penetration by an intrauterine device that mimicked endometriosis of the rectum [4]. Hemorrhoids are very common anorectal lesions [5, 6]. For women, pregnancy is a leading cause of hemorrhoids [5]. Anal masses and hematochezia are common symptoms of hemorrhoids [5]. In the current case, a patient with a history of hemorrhoids for 3 years came to our hospital for further diagnosis and treatment. During the digital rectal examination, in addition to anorectal hemorrhoids, a mass under the mucosa of the rectum was detected and confirmed as endometriosis by pathological examination. Without careful body examination, the hemorrhoids in this case might have masked the existence of endometriosis. Here, we focus on the diagnostic pitfalls for a relatively rare disease that presented in combination with a common disease.

## Case presentation

### Clinical history

A 37-year-old female was referred to our hospital for anal mass prolapse accompanied by bloody stools. Her symptoms started 3 years ago and continued until the time at which she was examined for this report. The patient had no abdominal pain, diarrhea, or weight loss. Prolapsus of the anus and rectum was detected by digital rectal examination. According to these findings, the patient was diagnosed with hemorrhoids. During the digital rectal examination, a mass of approximately 1.5 cm × 1.5 cm under the rectum mucosa 8 cm from the anus was also detected.

## Materials and methods

The tissue samples were examined by hematoxylin-eosin (HE) and immunohistochemistry staining as described previously [7]. The primary antibodies included actin (sm) (1:100, DAKO), CD10 (1:100, DAKO), CK (1:200, DAKO), Ki67 (1:100, DAKO) and ER (1:200, DAKO). Primary antibodies were omitted as a negative control. This study was approved by the institutional Ethics Committees of China Medical University and conducted in accordance with the ethical guidelines of the Declaration of Helsinki.

## Results

### Imaging and gross features

**Fig. 1:**
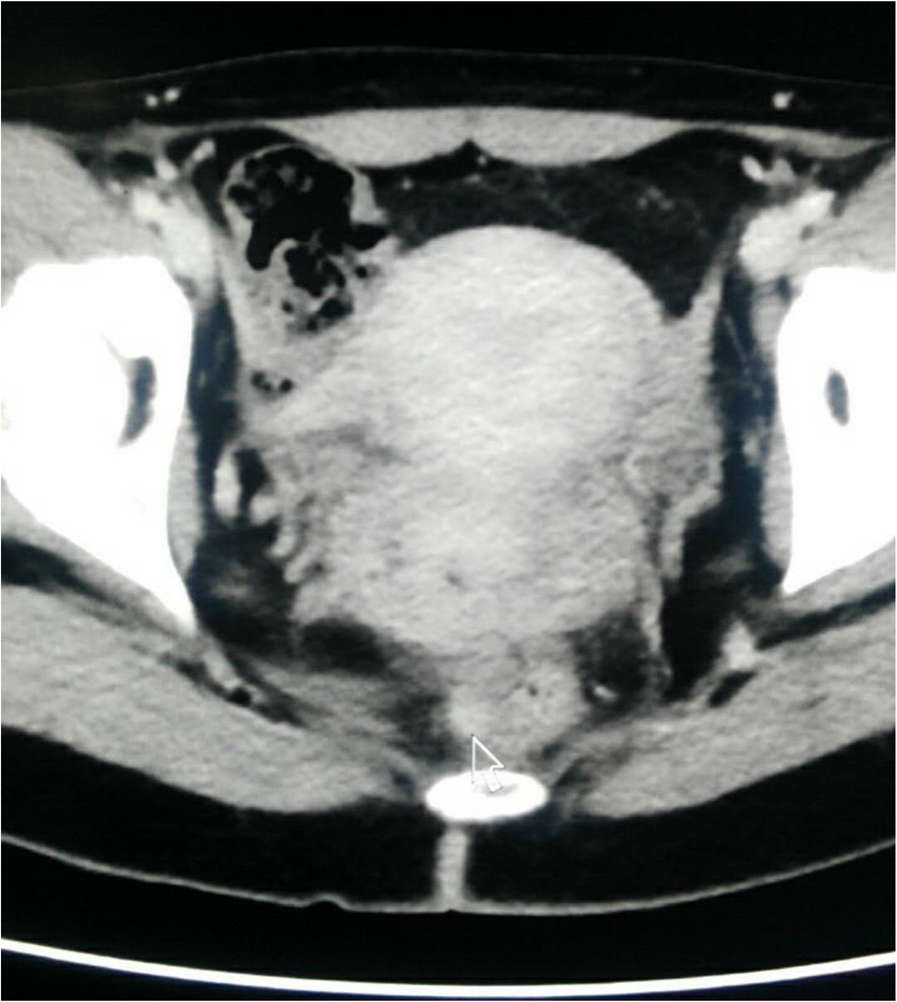
Imaging of the rectum. A dimly visible small nodule of approximately 9 mm (indicated by the arrow) was detected in the lower part of the left wall of the rectum. The walls of other gastrointestinal ducts were not markedly thickened. No obvious stenosis or expansion was found. No retroperitoneal enlarged lymph node was found

Abdomen computed tomography (CT) examination detected a mass in the wall of the rectum (Fig. 1). The nodule was approximately 9 mm and dimly visible. It was located in the lower part of the left wall of the rectum. No abnormity was detected in the walls of other gastrointestinal ducts. No retroperitoneal enlarged lymph node was found. On gross examination, the resected rectal wall was approximately 3 cm long, and the thickness was 0.9 cm. A mass of approximately 0.8 cm × 0.6 cm was found in the muscularis and submucosa of the rectum. The margin of the mass was not clear. The cut face was gray-white with scattered red hemorrhage.

### Microscopic features

**Fig. 2:**
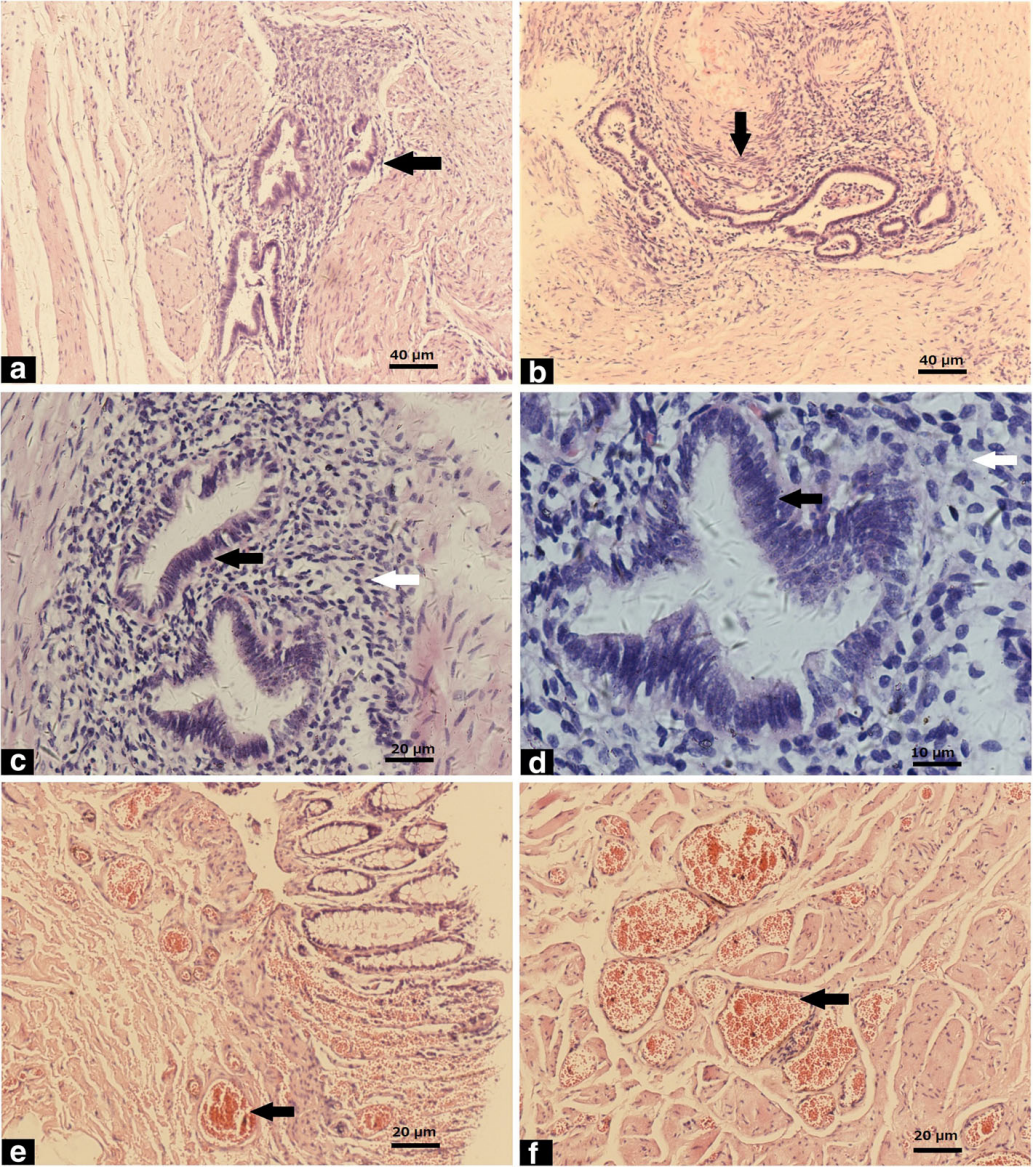
Histopathological findings. The ectopic endometrial tissues (black arrows) were mainly located in the muscularis (**a**) and submucosa (**b**) of the rectum. The ectopic endometrial glands (black arrow) and surrounding interstitial tissue (white arrow) at high magnification (**c**, **d**). The epithelial cells were columnar with no marked atypia (black arrow) (**D**). Dilated small vessels (black arrows) were also detected in the muscularis (**e**) and submucosa (**f**) of the rectum. Scale bar: A, B: 40 μm; C, E, F: 20 μm; D: 10 μm

The histopathological findings are shown in Fig. 2. The ectopic endometrial tissues were detected in the muscularis (A) and submucosa (B) of the rectum. The ectopic tissues included endometrial glands and surrounding interstitial tissue (C, D). The epithelial cells were columnar with no marked atypia and formed a monolayer (D). Dilated small vessels were also detected in the muscularis (E) and submucosa (F) of the rectum.

### Immunophenotype

**Fig. 3:**
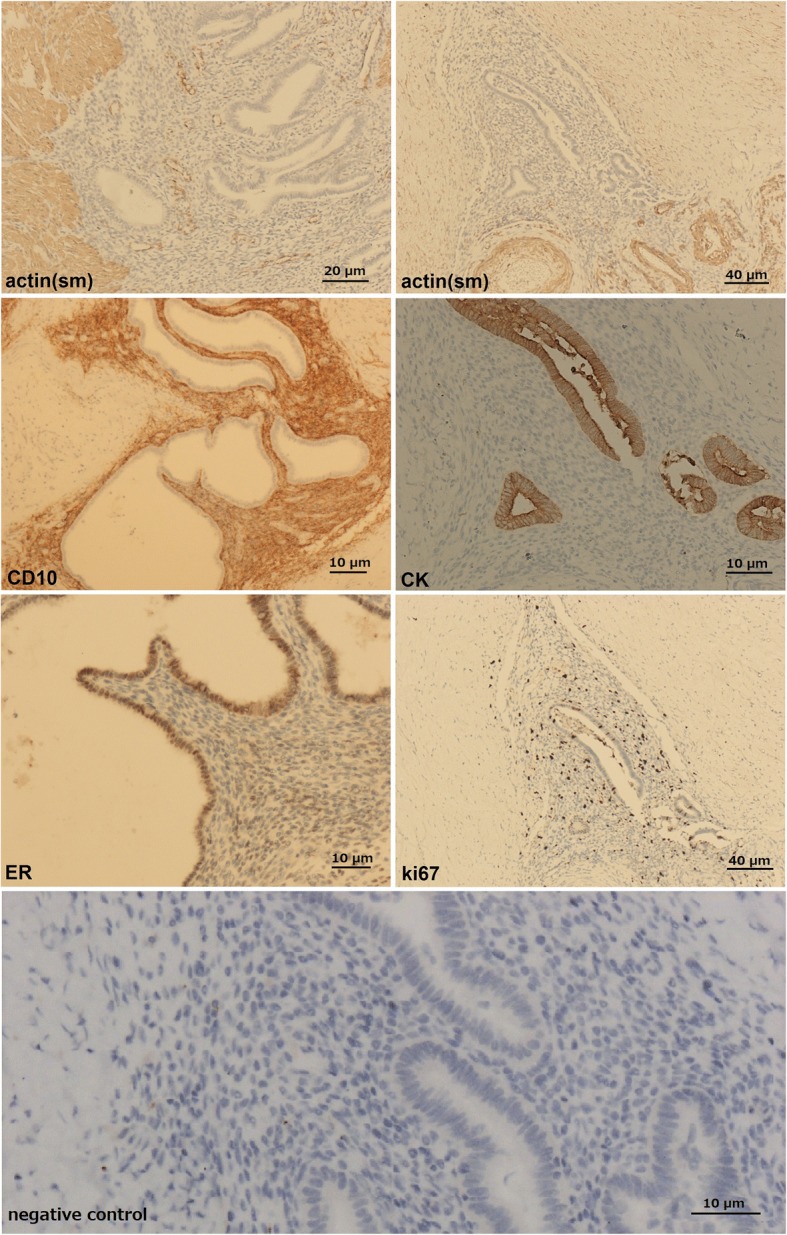
Immunostaining findings. Actin (sm) was positive in the smooth muscle cells (black arrow) in the muscularis of the rectum and negative in the submucosa (gray arrow) of the rectum and the endometrial interstitium (the white arrow). CD10 was positive in the endometrial interstitium (black arrow). CK and ER were positive in the epithelial cells of the endometrium (black arrows). The Ki67 index in the ectopic endometrial tissues was approximately 5% (black arrow). Negative control shows a representative negative control for CK immunostaining obtained by the omission of the primary antibody. Scale bar: actin (sm) (left), CK, CD10, ER, negative control: 20 μm; actin (sm) (right), Ki67: 40 μm

Immunostaining findings are shown in Fig. 3. Actin (sm) expression was observed in smooth muscle cells in the muscularis of the rectum. The submucosa of the rectum and the endometrial interstitium were negative for actin (sm). These actin (sm) staining findings can also confirm that the ectopic endometrial tissues were located in the muscularis and submucosa of the rectum. Positive CD10 staining confirmed the endometrial interstitium. CK and ER staining were found in the epithelial cells of the endometrium. A hot-spot was selected and 500 cells were counted and examined to calculate the Ki67 index. The Ki67 index in the ectopic endometrial tissues was approximately 5%.

## Discussion

Endometriosis occurring in the digestive tract mostly involves the rectum and sigmoid colon [1]. The peak age incidence is from 30 years old to before menopause [1]. Endometriosis of the large intestine is usually a component of pelvic endometriosis and secondary to the endometriosis of the rectovaginal septum [1]. Ectopic endometrial tissues in the rectum are typically located in the muscularis and rarely in the submucosa. The mucosa of the rectum is usually not involved [1]. In the current case, ectopic endometrial tissues were located in both the muscularis and submucosa of the rectum. Falleni reported a rare case of transmural endometriosis in the rectum, which was combined with primary adenocarcinoma [8]. In fast frozen section examination, endometriosis in the rectum may be mistaken for cancer for the glands seen in the muscularis of the rectum, which may be mistaken as cancer cell invasion. Adenocarcinoma can arise from endometriosis, including in the rectum [9]. The most common pathological type is endometrioid adenocarcinoma. Yu reported a rare case of clear cell adenocarcinoma arising from ectopic endometrial tissues in the rectum [9]. In the current case, the epithelial cells of the ectopic endometrial glands had no marked atypia and no malignant transformation were detected based on the microscopic histopathological findings.

Hematochezia is a common symptom in hemorrhoids and can also occur in endometriosis of the rectum [1, 10, 11]. The patient in the current case had the symptom of hematochezia for 3 years. The patient had prolapsus of the anus and rectum, which was easily detected by digital rectal examination. However, the endometriosis of the rectum was easily missed, as the lesion was under the mucosa of the rectum, and the site was quite far from the anus. In this case, the nodule of the endometriosis was quite small and difficult to detect. As the symptoms of hematochezia were fully consistent with hemorrhoids, a doctor might have overlooked the examination of the other parts of the rectum. Thus, the existence of hemorrhoids in the current case may represent a diagnostic pitfall for endometriosis. Stenosis of the intestine and rectal pain are common symptoms of endometriosis in the rectum [1]. Endometriosis in the rectum may be mistaken for cancer because of obstruction of the bowel canal revealed by colonoscopy and radiological examinations [3, 12]. It is worth noting that patients with endometriosis are relatively younger than those with intestine carcinoma. These symptoms were not observed in this case, which may be due to the relatively small size of the lesion. Because the specific symptoms of endometriosis in the rectum were not obvious in the current case, it was more easily missed, especially as it was combined with hemorrhoids. In summary, the reasons for diagnostic pitfalls for endometriosis in the rectum include (1) endometriosis combined with hemorrhoids. The patient had a 3-year history of hemorrhoids, and her symptoms were fully consistent with the diagnosis of hemorrhoids. (2) The nodule of the endometriosis in the rectum was small and not easy to detect. (3) Under the microscope, there was small vessel dilatation in the bowel with endometriosis, which could lead to a consideration of hemorrhoids unless extensive samples were taken. In summary, obvious symptoms can sometimes be misleading.

## Conclusion

Endometriosis of the rectum is relatively rare compared to hemorrhoids. When these diseases coexist, the rare disease is easily missed, especially when the common one causes obvious symptoms but the rare one does not. Another reason for the diagnostic challenge is that these diseases have some similarities in histopathological findings.

## Abbreviations

CT: Computed Tomography
HE: Hematoxylin-eosin
